# Effect of Chitosan Edible Coating on the Biochemical and Physical Characteristics of Carp Fillet* (Cyprinus carpio)* Stored at −18°C

**DOI:** 10.1155/2017/2812483

**Published:** 2017-05-28

**Authors:** Ana Gabriela Morachis-Valdez, Leobardo Manuel Gómez-Oliván, Imelda García-Argueta, María Dolores Hernández-Navarro, Daniel Díaz-Bandera, Octavio Dublán-García

**Affiliations:** ^1^Departamento de Toxicología Ambiental, Facultad de Química, Universidad Autónoma del Estado de México, Toluca, MEX, Mexico; ^2^Departamento de Alimentos, Facultad de Química, Universidad Autónoma del Estado de México, Toluca, MEX, Mexico; ^3^Departamento de Nutrición, Facultad de Medicina, Universidad Autónoma del Estado de México, Toluca, MEX, Mexico

## Abstract

The effect of an edible coating (EC) with 1.5% chitosan as an additive, on common carp* (Cyprinus carpio)* fillet, was determined evaluating the biochemical, physicochemical, textural, microbiological, and nutritional characteristics periodically during its storage in the freezer (−18°C), observing a decrease in the rate of biochemical reactions related to degradation (*p* < 0.05), hydroperoxides content (HPC) (0.8324 nM hydroperoxides/mg of protein versus 0.5540 nM/mg with regard to the EC sample), as well as protein carbonyl content (PCC) (0.5860 nM versus 0.4743 nM of reactive carbonyl groups/mg of protein of noncoated material), keeping properties for a longer period of time, and a lower protein solubility (7.8 mg of supernatant protein/mg of total protein versus 6.8 mg/mg) and less loss of moisture (8% less, with regard to EC); for the nutritional characteristics of the fillet, lysine is the limiting aminoacid in the sample without EC, while leucine is the limiting aminoacid for the EC sample. According to microbial growth, the count was 2.2 × 10^5^ CFU/g of sample in mesophiles versus 4.7 × 10^4^ in the EC sample. The results indicate that the use of EC added with chitosan maintains the quality of the product regarding lipid and protein oxidation until fourth month of storage, maintaining moisture content without variation for at least 3 months, and inhibits microbial growth up to 2 logarithmic units, during five months of frozen storage.

## 1. Introduction

The quality of fish is a complex concept, in which nutritional, microbiological, biochemical, and physicochemical attributes are involved. The freshness of fish decreases after its sacrifice; this is due to microbiological contamination and various biochemical reactions which produce changes in the protein fractions [1]. Some investigations have emphasized how the lipid compounds are altered due to oxidative deterioration [2]. Proteins including the sarcoplasmic, myofibrillar, and stromal proteins are susceptible to oxidative damage by intermediates of lipid oxidation [4-hydroxy-trans-2-nonenal (HNE), acrolein, malondialdehyde (MDA), glyoxal, and 4-oxo-trans-2-nonenal (ONE)], isoketals and metallic ions (such as the iron in the heme group or the copper and zinc found in enzymes and metalloenzymes) present in the muscles of animals, and those originated through processing (exposure of meat to oxygen, light, and temperature, cooling, use of additives, irradiation, and vacuum-packaging) that initiate oxidative damage, generating changes in flavor, color, texture/structure, and nutritional value [1–5]. The use of low temperatures such as freezing is a general method used for the control and decrease of biochemical changes that can occur during storage time; however, this does not completely inhibit the microbiological and chemical reactions that result in the deterioration of the quality of the fish, for which the use of edible coatings (EC) as adjuvants of preservation have demonstrated to provide an increase in shelf-life, due to their function as a barrier to oxygen, besides being employed as a vehicle of diverse components such as essential oils, bacteriocins, organic acids, and chitosan, which help in the control of oxidation and diminish the deterioration by microorganisms [6–8]. Chitosan (poly-b-(1–4)-D-glucosamine) is a versatile biopolymer, having a broad range of applications in the food industry. It has been reported to have a number of functional properties that make chitosan useful in food preservation; these include its antimicrobial activity [2] and antioxidant activity [9] and its ability to form protective films or coatings [10]. Although studies have been carried out concerning the use of chitosan as an antioxidant and/or an antimicrobial agent in EC [3, 9–11], none have thoroughly discussed its effect on nutritional properties. The purpose of this study was the use of an EC containing 1.5% chitosan in order to reduce the speed of deterioration caused by oxidation and/or microbial growth, evaluating physicochemical, textural, and nutritional properties during storage at commercial freezing temperatures (−18°C) in common carp.

## 2. Materials and Methods

### 2.1. Preparation and Treatment of Fish Samples

#### 2.1.1. Chemicals

Chitosan, medium molecular weight, deacetylation value of 75–85%, and viscosity of 200–800 cP, was purchased from Aldrich Chemical Co.

Bovine serum albumin, acrylamide, N,N′-methylenebisacrylamide, trichloroacetic acid (TCA), FeSO_4_, sulfuric acid, cumene hydroperoxide (CHP), butylhydroxytoluene, methanol, xylenol orange, di-nitrophenylhydrazine (DNPH), guanidine, ethanol, ethyl acetate, hydrochloric acid, Coomassie® Brilliant Blue R-250, thioglycolic acid, NBD-Cl, and o-phthalaldehyde (OPA) were purchased from Sigma-Aldrich (St. Louis, Missouri, USA).

Thiobarbituric acid was purchased (TBA) from Fluka (Sigma-Aldrich, Toluca, MX); sodium chloride, EDTA disodium salt, N,N′,N′- tetramethylethylenediamine (TEMED), Tris (base), urea, *β*-mercaptoethanol, glycine, acetic acid glacial, sodium phosphate monobasic, sodium phosphate dibasic, and copper sulfate pentahydrate were purchased from J.T. Baker (Pennsylvania, USA); sodium carbonate and lactic acid were purchased from Fermont (Monterrey, MX); sodium dodecyl sulfate (SDS) and bromophenol blue were obtained from Hycel (Mexico, MX); and plate count agar was purchased from Bioxon, Becton and Dickinson (Mexico, MX). All reagents used were of analytical grade.

#### 2.1.2. Fish Sample Preparation

A total of 40 freshwater carps* (Cyprinus carpio)*, with an average weight of 550–650 g, were purchased at Tiacaque Aquacultural Center in Toluca, State of Mexico, Mexico, and were transferred to the Food Science Laboratory in the School of Chemistry, at the Universidad Autónoma del Estado de México, and were filleted by hand using knives and cutting boards sanitized in chlorine solution and rinsed in sterile distilled water. The fish were harvested during May 2016. Two fillets were obtained from each fish after removing the head and bone and were then immersed in the coating solution.

#### 2.1.3. Preparation of Coating Solution and Treated Fillets

Chitosan solution was prepared with 1.5% (w/v) chitosan in 1% v/v lactic acid. To achieve complete dispersion of chitosan, the solution was stirred at 40°C for 1.5 h, on a hotplate/magnetic stirrer; the final coating forming solution consisted of 13% whey, 6% gelatin, 13% glycerol, and 4% inulin, according to Garcia-Argueta et al. [12], with a final pH of 3.5. Fillet samples were randomly assigned to two treatment batches consisting of one control batch (uncoated) and one batch treated with the coating solution. For each coated batch, approximately 20 carp fillets (12–15 cm) were immersed for 15 s in the coating solution and then allowed to stand for 1 min. Then, the fish fillets were drained on a sterile metal net and air-dried for 20 min in order to form the edible coatings, placed on polyethylene containers, and then stored at −18 ± 1°C for subsequent quality assessment in a commercial freezer (Torrey, México, MX). Chemical and microbiological analyses were performed at monthly intervals to determine the overall quality of fish, for five months.

### 2.2. Chemical Analyses

#### 2.2.1. Moisture and Total Protein Analysis

Moisture content was determined by difference in weight between the fresh sample of minced fillet and the dried sample after drying in an oven at 105°C until reaching constant weight. The result is expressed as a percentage of moisture. The content of total protein was determined through the Kjeldahl method and results are expressed as g of protein/100 g of fish, as described in AOAC [13].

#### 2.2.2. pH

10 g of fillet muscle was weighed and homogenized at high speed in mixer/blender (Osterizer 450-20) for 1 min with 90 mL of distilled water. Connective tissue was eliminated by filtering with cloth, in accordance with that described by Owen et al. [14]. pH was determined with a digital pH meter (Conductronic pH 120, New York, USA).

#### 2.2.3. Myofibrillar Protein Extraction

Myofibrillar protein (MP) was obtained in accordance with the methodology described by Ngapo et al. [15], with slight modifications. 100 g of common carp muscle was homogenized with a blender for 10 min with a mixture of ice-cold water 1 : 1 : 1 (w/w/v) and was then placed in an ice bath with a magnetic stirrer. The myofibrillar suspension was filtered through two layers of cloth in order to remove the connective tissue; this procedure was carried out twice. The homogenized muscle was then centrifuged at 3000 ×g at 4°C for 25 min and the supernatant was discarded. The protein concentration of the myofibrillar precipitate was determined using the Biuret method [16]. 25 mg/mL of MP was stored in a glass container with a lid for the formation of the gel. Gel forming was developed in two-step heating, first, incubation at 40°C for 30 min followed by heating with a gradual increase until 90°C was reached with constant stirring and then maintaining it for 20 min. Finally glass containers were removed and stored at 4°C.

#### 2.2.4. Solubility

According to Pilosof [17], 2 g of MP was centrifuged at 2500 ×g at 4°C for 30 min. The protein content in the supernatant was determined, as well as the total protein content in the MP sample prior to centrifugation. Solubility was defined by following equation:

(1) $$\begin{array}{c} \text{Solubility}=\frac{\text{Protein content in supernatant}}{\text{Protein content in the sample}}\times 100. \end{array}$$

#### 2.2.5. Total Sulfhydryl Content

The total content of sulfhydryls (SH) was determined according to the method described by Ellman [18]. An aliquot of 1 mL of MP solution (5 mg/mL) reacted with 9 mL of Tris-glycine buffer (10.4 g of Tris-HCl, 6.9 g of glycine, 480 g of urea, and 1.2 g of EDTA/L at pH 8.0) at room temperature for 30 min. 0.05 mL of Ellman reagent (4 mg DTNB/mL) was added to aliquots of 3 mL and was incubated in darkness for 30 min. The reaction mixture was measured at 412 nm using a TU-1800 spectrophotometer (Beijing Purkinje General Instrument Co. Ltd., Beijing, China). The concentration of SH was expressed as total *μ*M SH/mg of protein.

#### 2.2.6. Determination of Total Volatile Base (TVB-N) Content

The content of TVB-N was determined according to the Conway and Byrne method [19], with slight modifications. 5 g of the homogenized sample was added to 4% TCA in a 1 : 2 (w/v) ratio. Then, it was filtered through Whatman Number 1 paper (Schleicher & Schuell, Maidstone, England). 1 mL of the filtrate obtained was placed in the outer ring of the Conway Camara, while, in the inner ring, a solution of 1% boric acid containing Shiro Tashiro indicator was added. To initiate the reaction, 2 mL of K_2_CO_3_ was mixed with the filtrate. The camera was incubated at 25°C for 24 hr. The solution of the inner ring was titrated using 0.01 N HCl until a change in the color to a pink tone.

#### 2.2.7. Determination of Hydroperoxides (HPC)

The content of HPC was determined by the Jiang et al. method [20] (FOX—ferrous oxidation-xylenol orange). A 100 *μ*L aliquot of supernatant was obtained by the deproteinization of the sample with 10% TCA. 900 *μ*L of the reaction mixture was added, consisting of 25 mM H_2_SO_4_, 0.25 mM FeSO_4_ 0.1 mM, xylenol orange, and 4 mM butyl hydroxytoluene in 90% (v/v). The mixture obtained was incubated for 60 min at room temperature and absorbance was measured at 560 nm against the reaction blank in the spectrophotometer. The results were interpolated in a normal curve previously elaborated and were expressed as nM HPC/mg protein.

#### 2.2.8. Determination of Lipoperoxides (LPX)

For the determination of LPX, the technique described by Büege and Aust thiobarbituric acid reactive substances (TBARS) was employed, which consists of an aliquot of 100 *μ*L of supernatant, obtained with prior deproteinization, that was added until 1 mL of Tris-HCl buffer solution pH 7.4 is reached. The samples were incubated at 37°C for 30 min; then 2 mL of the TBA-TCA reagent (0.375% TBA in 15% TCA) was added and thoroughly mixed using a vortex. It was taken to boiling point in a hot water bath for 45 min and was left to cool, eliminating the precipitate formed by centrifugation at 3000 ×g for 10 min. Absorbance readings were carried out at a wavelength of 535 nm against a reaction blank. The content of malondialdehyde (MDA) was calculated utilizing the molar extinction coefficient (MEC) of MDA (1.56 × 10^5^ M/cm). The results were expressed as mM MDA/mg protein.

#### 2.2.9. Determination of Protein Carbonyl Content (PCC)

The method is described by Levine et al. [21] and modified by Parvez and Raisuddin [22] and Burcham [23]. To an aliquot of 100 *μ*L of supernatant obtained from the deproteinized sample, 150 *μ*L of 10 mM DNPH dissolved in 2 M HCl was added, allowing for the reaction to be carried out in the dark for an hour at room temperature, placing 500 *μ*L of 20% TCA and placing the mixture at rest for 15 min at 4°C. The sample was centrifuged at 11,000 ×g for 5 min. The precipitate obtained was washed at least three times with a solution of ethyl acetate : ethanol (1 : 1). Using a 6 M guanidine (pH 2.3) solution, the button was dissolved and was incubated for 30 min at 37°C. Absorbance readings were obtained at 366 nm, employing the corresponding MEC of 21,000 M/cm. The results were expressed as nM reactive carbonyls formed (C=O)/mg protein.

#### 2.2.10. SDS-PAGE

SDS gel electrophoresis was carried out according to Laemmli [24], with slight modifications in electrophoresis equipment, which consists of Bio-Rad Mini-PROTEAN II Cell camera, employing 10% acrylamide. MP extracts were added to 10% urea and buffer sample [0.1 M Tris-HCl (pH 6.8), 0.4% SDS, 10% glycerol, and 0.004% bromophenol blue]. The gel of 140 × 140 nm was prepared at a* T* = 10% in 1.2 M Tris-HCl (pH 8.8) and 0.3% SDS; the concentration gel at a* T* = 4% was prepared with 0.25 M Tris-HCl (pH 6.8) and 0.2% SDS. The electrode buffer contained 0.025 M Tris-HCl, 0.192 M glycine, and 0.15% SDS at pH 8.16. An electrophoretic run was carried out with a current of 200 volts; once the run was concluded, the gels were stained with a solution consisting of 40% methanol, 15% acetic acid, and 0.1% Coomassie R-250 Brilliant Blue.

#### 2.2.11. Amino Acid Composition

3 mg of the dehydrated simple was placed into tubes to carry out hydrolysis, with 6 N HCl and thioglycolic acid as antioxidants. Posteriorly, test tubes were heated for 6 hr at 150°C. At the end of hydrolysis, the reagent was evaporated in a Buchi rotary evaporator (Buchi, Flawil, Switzerland), obtaining a concentrate which was resuspended in 2 mL of 0.2 N sodium citrate buffer, pH 2.2.

For the determination of primary aminoacids, 250 *μ*L of hydrolyzed extract was taken and mixed with 250 *μ*L of o-phthalaldehyde (OPA); an aliquot of 20 *μ*L was injected into the HPLC chromatograph (Varian 9012). For the determination of secondary aminoacids (proline and hydroxyproline), 125 *μ*L of the lyophilized extract was put in 0.5 mL of 0.4 M borate buffer, pH 10.4. From this solution, 250 *μ*L was taken and mixed with 250 *μ*L of the derivative solution (NBD-Cl, 2 mg/mL, in MeOH); once filtered, the solution was heated to 60°C during 5 min in a dark vial with a lid. The derivatization reaction was stopped with the addition of 50 *μ*L of 1 M HCl and was cooled to 0°C. For the analysis, 20 mL of the final extract was taken and injected into the HPLC; a 10 cm × 4.6 mm × 3 *μ*m Varian Microsorb C18 column was utilized for this analysis, as was HPLC-grade methanol (with 99% purity) and sodium acetate buffer (pH 7.2) as mobile phase. A 430020-02 Fluorichrom fluorescence detector was used, and the quantification was carried out using external standards as reference.

### 2.3. Total Viable Counts (TVC)

A 10 g sample was homogenized in 90 mL of 0.1% peptone solution. Decimal dilutions were prepared from this solution and plated using plate count agar. The inoculated plates were incubated at 35°C for 48 h for total viable counts (log⁡10 CFU/g), as described by Ibrahim Sallam [25].

### 2.4. Statistical Analysis

All experiments were performed in triplicate and a completely randomized design was used. All data were statistically analyzed by SPSS/PC software (version 17). One-way analysis of variance, independent sampling, and paired Student's *t*-tests were used for comparison of the means.

## 3. Results and Discussion

### 3.1. Physicochemical Analysis

#### 3.1.1. Moisture

**Figure 1:**
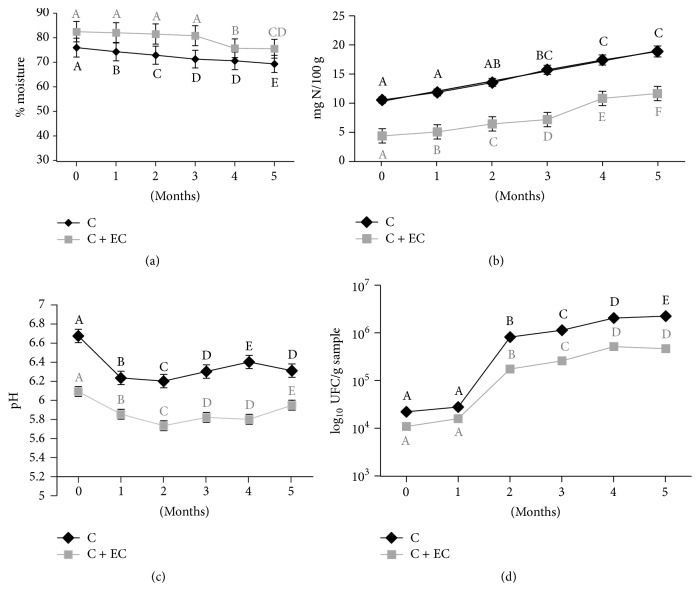
Changes in moisture (a), total volatile basic nitrogen TVB-N (b), pH (c), and total viable count (TVC) (d) values of common carp fillets stored at −18°C for 5 months. The results are the mean of three replications. C: fillet carp without coating; C + EC: fillet carp with edible coating. The different letters indicate significant differences between treatment times for the same treatment (*p* < 0.05).

During its storage under freezing, a significant (*p* < 0.05) decrease in moisture in both treatments was observed (Figure 1(a)). For the carp with the coating, the decrease was observed until the fourth month; this could be due to the chitosan in the EC, which might promote cross-linking in the gelatin, thus diminishing the free volume of the polymeric matrix, which reduces the diffusion rate of the water molecules through the coating film. The aforementioned results in a decrease in vapor permeability in the fillet, as well as with the coating itself [26]. According to Dutta et al. [8], this is a desirable characteristic in coatings and is not observed in the control sample of this study, since a loss in moisture occurs (*p* < 0.05) during all of its storage period. After five months of storage, the percentage of loss was similar in both the control sample and the sample with the treatment; this could be due to the fact that both treatments were stored in polyethylene containers, which act as protection, where the coating would be able to maintain the moisture of the product during the first four months of storage [27].

#### 3.1.2. pH

A significant decrease (*p* < 0,05) in pH was observed (Figure 1(c)) at the end of both treatments, which could be associated with the production of lactic acid through anaerobic glycolysis and the liberation of inorganic phosphate, a product of ATP degradation. An increase in pH is observed by the second month for the control sample and by the third month for the coated sample, which could be due to the accumulation of basic compounds such as ammonia and trimethylamine, a result of autolytic and microbial reactions [4, 28–30]. The greatest variation in percentage at the end of storage corresponds to the noncoated sample, a lower pH in the sample with the coating can bolster microbial inhibition and contribute to the preservation of the samples inhibiting the endogenous proteases, and this result suggests that, during storage, the coating diminished the decrease in pH [11, 28].

#### 3.1.3. Total Volatile Basic Nitrogen (TVB-N)

TVB-N is a group primarily composed of primary, secondary, and tertiary amines which are used as indicators of meat deterioration; the increase in these is related to the activity of endogenous enzymes and bacteria [28]. According to Connell [31] and Giménez et al. [32], 25–40 mg of N/100 g of tissue is considered as acceptable for consumption [9, 11, 28]. Although both samples have values below said limit (Figure 1(b)), the concentration of TVB-N is greater in the control sample in each stage of storage; this could be due to the fact that the presence of chitosan helps in reducing the capacity of bacteria for oxidative deamination of nonprotein nitrogenated compounds [11, 28]. The storage time was not enough to identify when the acceptable threshold is exceeded, due to the fact that enzymatic and microbial activity diminish at low temperatures. The carp utilized in this study presented adequate conditions for human consumption at the end of the storage period, coinciding with that reported by Soares et al. [27].

### 3.2. Microbiological Changes

#### 3.2.1. Total Viable Count

Microbial activity is a limiting factor in the quality of the fish, and the total viable count has been used as indicator of acceptability of the same [29, 33]. The initial value for the carp without the coating was 2.3 log⁡10^4^ CFU/g and 1.1 log⁡10^4^ CFU/g for the carp with the coating; these values depend on the environment from which the fish is obtained as well as postmortem conditions [11]. The evolution of the aforementioned is detailed in Figure 1(d), observing significant differences (*p* < 0.05) by the second month, obtaining a time-dependent increase in both treatments; however, the limit recommended by the ICMSF, [34] of 5 × 10^5^ CFU/g, for quality fish, is not exceeded [29]. The properties of the chitosan added to the coating had an inhibitory effect, thereby obtaining 4.7 log⁡10^4^ CFU/g, while, for the control, a value of 2.2 log⁡10^6^ CFU/g was obtained after five months of storage. The antimicrobial effect of this compound has been widely reported by Fernández-Saiz et al. [35], Jeon et al. [36], and López-Caballero et al. [37], and its mechanism of action is related to the rupture of the lipopolysaccharide layer of the external membrane of Gram-negative bacteria and its function as a barrier against the transfer of oxygen. Another mechanism of action could be the interaction with anionic groups on the cell surface, due to its polycationic nature [3, 9]; this demonstrates that the EC inhibits microbial growth up to 2 logarithmic units, decreasing reactions involved in deterioration by microorganisms during its storage in the freezer.

### 3.3. Lipid Oxidation Products

**Figure 2:**
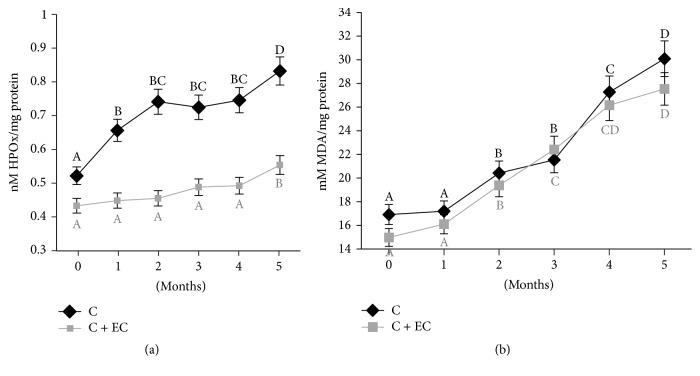
Changes in HPOx content (a) and MDA content (b) values of common carp fillets stored at −18°C for 5 months. The results are the mean of three replications. C: fillet carp without coating; C + EC: fillet carp with edible coating. The different letters indicate significant differences between treatment times for the same treatment (*p* < 0.05).

The concentration of primary products of oxidation can be measured by the content of peroxides. The carp with coating shows a significant increase (*p* < 0.05) in peroxides by the fourth month and presents a final value of 0.55 nM HPOx/mg of protein (Figure 2(a)) while the control sample presents an increase by the first month, having an increase of 59% with regard to the sample with coating; this demonstrates that the coating retards lipid oxidation in the carp fillet. These results are in accordance with Ojagh et al. [9], Nowzari et al. [10], Jeon et al. [36], and Li et al. [38] and those that report that additional coatings with chitosan retard production of oxidated primary compounds in herring, trout, cod, and croaker in freezer storage as well as in ice storage. Lipid oxidation in fish is influenced by various factors like fat content, the degree of microsome associated with the oxidation system, heme group content, and the presence of ions [10, 38]. Chitosan-added coatings act as excellent barriers to the permeability of oxygen, once they are applied directly over the meat's surface, retarding the diffusion of oxygen [10].

In storage at freezing temperature (−18°C), oxidation is the most important factor in deterioration, even over microbial activity. TBARS quantifies the compounds responsible for the loss of flavor and scent and is also important in the stages of deterioration of foods. The value of TBA is an indicator of lipid oxidation widely used, which quantifies the content of malondialdehyde (MDA), formed from hydroperoxides, which are the initial products of the oxidation of unsaturated fatty acids by oxygen [29, 39]. In the present study, the values of TBA of both treatment samples presented a significant increase (*p* < 0.05) by the second and forth months; however, by the fourth month the control sample presented a 39% increase with regard to a 6% increase in the sample with coating; this is due to the absence of chitosan in the control sample's coating. This same behavior was observed by Jeon et al. [36], in herring and cod with a chitosan coating, and Ojagh et al. [9] in rainbow trout. The antioxidative mechanism of the chitosan is due to the fact that its primary amino groups form a stable fluorosphere with volatile aldehydes such as malondialdehyde, derived from the rupture of fats during oxidation [38].

The results indicate that the chitosan employed in a 1.5% EC preserves the fish fillet through reduction of lipid oxidation.

### 3.4. Products of Protein Oxidation

**Figure 3:**
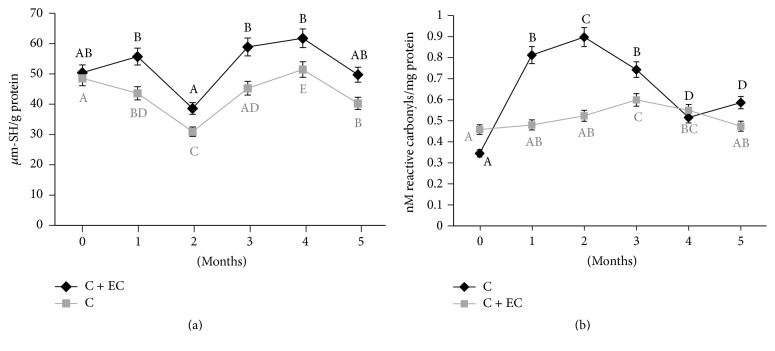
Changes in sulfhydryl content (a) and protein carbonyl content (b) values of common carp fillets stored at −18°C for 5 months. The results are the mean of three replications. C: fillet carp without coating; C + EC: fillet carp with edible coating. The different letters indicate significant differences between treatment times for the same treatment (*p* < 0.05).

Protein oxidation in carp fillet during freezer storage is shown in Figure 3; there was a significant increase (*p* < 0.05) in the formation of carbonylated proteins during storage in the control sample by the first month, while the sample with coating presented an increase until the third month and does not present differences at the end of storage. The increase was from 0.35 to 0.89 nm/mg of protein for the control sample and from 0.45 to 0.59 nm/mg of protein at maximum oxidation point. The samples of processed fillet include red and white muscle, and thus it is possible to find a high proportion of heme proteins (hemoglobin and myoglobin) and, consequently, a concentration of iron, in addition to what can be found chelated to proteins, favoring protein and lipid oxidation. By the fourth month, both samples present a reduction in the concentration of carbonyl groups, which has been reported by other authors during a storage at −20°C, suggesting interactions between carbonyls and other cell components, for which the formation of carbonyl groups should be considered as a step in oxidation processes and not as a sole, stable marker of protein oxidation [40]. The oxidation of proteins is associated with the decrease in sulfhydryl groups, which are converted to disulfides. During freezer storage, a significant decrease (*p* < 0.05) was observed during the second month in both samples. Proteins are attacked by reactive oxygen species (ROS), where its creation in fish is due to diverse external factors, such as noise, manipulation, slaughter, or the presence of metals such as iron. The interaction can lead to the formation of carbonyl groups and the loss of sulfhydryl groups [3, 40], affecting structural, functional, and nutritional properties, for which the use of EC with chitosan could reduce mentioned effects.

### 3.5. Aminoacid Content

The protein content in fresh carp is similar to that reported by FAO (21%), in frozen species, as well as frozen species but with coating (Table 1). The protein of fish is considered of the highest quality compared to standard proteins reported by FAO and although the information concerning its nutritional value is widely known, few are the studies that refer to its composition, the scarcest being in common carp [41]. It is known that the content and bioavailability of aminoacids can be affected by different operations such as drying, fermentation, extrusion, and even germination [42]. In this study, it was observed that the aminoacid content in carp suffered a significant decrease (*p* < 0.05) after 5 months of storage due to freezing, while the species with the coating only presented a decrease in the levels of Cys, His, Tyr, Thr, Met, and Lys; this could be due to protein oxidation by intermediaries of lipid oxidation and environment factors such as pH, temperature, water activity, and the presence of promoters and inhibitors like phenolic compounds [43]. The sulfur aminoacids such as Met and Cys are highly susceptible to oxidation in the presence of oxidized lipid products which lead to the formation of a variety of compounds such as sulphone, sulfoxides, and disulfide derivatives [44]. In the present study, a decrease in Met and Cys can be observed in the frozen fillet with regard to the control. Once the edible coating is added, a protective activity can be observed for Cys (0.84 g/100 g in frozen fillet and 0.91 g/100 g in coated fillet); this can be due to cross-linking, which is usually attributed to the formation of Cys (disulfide bonds) and dityrosines (from two Cys and two Tyr residues) [45]. The loss of Thr and Lys could be due to the formation of intermediary adducts such as *α*-amino-3-keto butyric acid and *α*-amino adipic semialdehyde, as a consequence of oxidation catalyzed by metals from the metalloproteins of the food matrix [43].

Common carp protein is characterized by a high content of essential aminoacids, compared to the standard protein established by the FAO, exceeding values in the case of Met + Cys and Val (Table 2) in fresh carp, having Leu as the limiting aminoacid and presenting a “limiting aminoacid” index (or chemical index) of 82.27; this index is a basic parameter used for the evaluation of the nutritional value of a food, which refers to the minor content of an essential aminoacid with regard to a given standard protein, called the “limiting aminoacid.” In this study, the essential aminoacids in the sample in freezing without EC decreased, only maintaining Val as the excess aminoacid and having Lys as the limiting aminoacid. This change in the limiting aminoacid could be a consequence of enzymatic reactions and/or bacterial growth during storage [46]. On the other hand, the frozen carp with EC continued to present a higher concentration in the aforementioned aminoacids (Iso, Met + Cys, and Val), thereby presenting a protective effect in Lys, which results in great benefit since fish could be maintained as an important source of this aminoacid after 5 months of storage in the freezer [42, 47]. The chemical index diminishes 11 units in the fillet without EC and 3 units for the fillet with the EC after freezing, which suggests that EC has a protective effect over essential aminoacids.

### 3.6. SDS-PAGE

**Figure 4:**
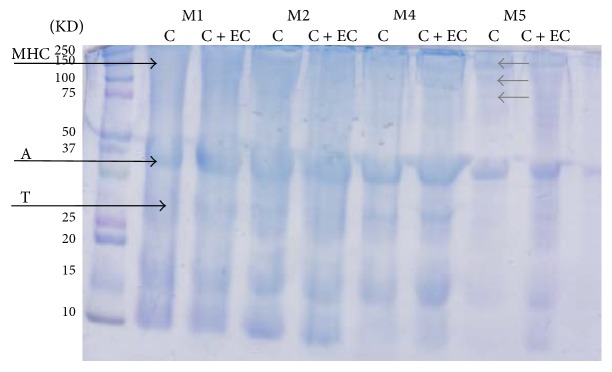
SDS-PAGE of common carp fillets stored at −18°C; control batch (C) fillet without coating and filleted carp with edible coating (C + EC); M1, M2, M4, and M5 from first to fifth month during storage.

The molecular weight profiles obtained through SDS-PAGE of the MPs extracted from the different treatments during storage are shown in Figure 4, in which the composition of the MPs of common carp was myosin heavy chains (MHC), actin (A), and troponin (T), observing that the sample without coating presents, after 5 months of storage, molecular weight bands lower than those of myosin, with an interval of 150, 100, and 75 kDa, approximately; this is probably due to carbonylation of MHC, coinciding with that reported by Kjærsgård et al. [48] in rainbow trout. Likewise, Passi et al. (2005) reported an increase of oxidized proteins in different species of Mediterranean fish after lipid oxidation, possibly due to the presence of the heme group in myoglobin [49]. For the samples with coating, no notable changes were observed in the SDS-PAGE profile, which could suggest that the coating is retarding the oxidation mechanism, showing that actin was the least oxidized during freezing, coinciding with that reported by Eymard et al. [40]. In spite of the high susceptibility of myofibrillary proteins to oxidation, edible coatings could help in the preservation of protein integrity of aquatic/marine species stored during freezing, keeping functional and nutritional properties for more time.

## 4. Conclusions

The use of EC added with chitosan allows retention of the physicochemical and nutritional characteristics of the common carp for more time during storage, diminishing the loss of moisture, bacterial growth, nutritional value, and speed of lipid and protein oxidation in oxidation products (hydroperoxides, lipoperoxidation, and carbonyl proteins), as well as an indication of the aminoacids present in the common carp fillet with EC, being an excellent alternative as an adjuvant in the conservation through freezing of aquatic species of economic importance on a global scale.

## Figures and Tables

**Table 1:** Aminoacid composition of common carp fillet proteins, 5-month frozen carp, and 5-month frozen carp + edible coating, stored at −18°C.

Aminoacids	Carp	5-month frozen carp	5-month frozen carp + edible coating
Asparagine	6.28 ± 0.18^a^	5.03 ± 0.12^b^	5.98 ± 0.22^a^
Glutamic acid	10.75 ± 0.22^a^	8.53 ± 0.18^c^	9.05 ± 0.15^b^
Alanine	4.98 ± 0.18^a^	3.76 ± 0.15^c^	4.28 ± 0.23^b^
Arginine	3.74 ± 0.10^a^	3.11 ± 0.12^b^	3.45 ± 0.18^a^
Cysteine	0.98 ± 0.07^a^	0.84 ± 0.08^b^	0.91 ± 0.06^a^
Phenylalanine	3.51 ± 0.24^a^	2.71 ± 0.28^b^	3.29 ± 0.19^a^
Glycine	3.34 ± 0.12^a^	2.76 ± 0.19^b^	3.11 + 0.16^a^
Histidine	2.48 ± 0.08^a^	1.5 ± 0.05^c^	2.01 + 0.12^b^
Isoleucine	3.89 ± 0.24^a^	2.31 ± 0.22^b^	3.28 + 0.17^a^
Leucine	5.43 ± 0.32^a^	4.76 ± 0.38^b^	5.21 ± 0.34^a^
Lysine	5.53 ± 0.28^a^	4.12 ± 0.22^b^	5.3 ± 0.19^c^
Methionine	2.73 ± 0.08^a^	1.08 ± 0.07^b^	2.06 ± 0.09^c^
Serine	2.72 ± 0.07^a^	1.97 ± 0.08^c^	2.25 ± 0.11^b^
Tyrosine	2.67 ± 0.15^a^	1.86 ± 0.21^b^	2.22 ± 0.13^c^
Threonine	3.16 ± 0.22^a^	2.42 ± 0.12^b^	3.08 ± 0.15^a^
Valine	4.28 ± 0.19^a^	3.76 ± 0.32^b^	4.12 ± 0.23^a^
Protein (%)	22.39 ± 1.7^a^	22.65 ± 1.2^a^	22.87 ± 1.4^a^

^a, b, c^ *p* < 0.05.

**Table 2:** Values of the limiting aminoacid index (%).

Aminoacids	Standard FAO/WHO (1991)^c^	Carp		5-month frozen carp		5-month frozen carp + edible coating	
		g/100 g protein	%	g/100 g protein	%	g/100 g protein	%
Phe + Tyr^a^	6,30	6,18	98,10	4,57	72,54	5,51	87,46
Isoleucine	2,80	3,89	138,93	2,31	82,50	3,28	117,14
Leucine	6,60	5,43	**82,27**	4,76	72,12	5,21	**78,94**
Lysine	5,80	5,53	95,34	4,12	**71,03**	5,30	91,38
Met + Cys^b^	2,50	3,71	148,40	1,92	76,80	2,97	118,80
Threonine	3,40	3,16	92,94	2,42	71,18	3,08	90,59
Valine	3,50	4,28	122,29	3,76	107,43	4,12	117,71
Amino acid index			82,27		71,03		78,94

^a^Phenylalanine + tyrosine; ^b^methionine + cysteine; ^c^according to Usydus et al. [47].
